# Risk factors for failing sub-Tenon’s triamcinolone acetonide for uveitic macular edema

**DOI:** 10.1186/s12348-024-00386-1

**Published:** 2024-02-01

**Authors:** Amit K. Reddy, Jennifer L. Patnaik, Alan G. Palestine

**Affiliations:** Department of Ophthalmology, University of Colorado School of Medicine, 1675 Aurora Court, F731, Aurora, CO 80045 USA

**Keywords:** Uveitis, Uveitic macular edema, Corticosteroid injections, Sub-tenon’s triamcinolone acetonide, Intravitreal corticosteroids, Intravitreal dexamethasone implant

## Abstract

**Background:**

Sub-Tenon’s triamcinolone acetonide (STA) is less effective than intravitreal corticosteroids in the treatment of uveitic macular edema (ME), but does have some relative advantages, including substantially lower cost and decreased risk of post-injection ocular hypertension. It would be useful for clinicians to know which eyes may respond well to STA and not necessarily require intravitreal therapy. The objective of this study is to identify risk factors for failing STA for the treatment of uveitic ME.

**Main body:**

A retrospective cohort study was performed. Medical records were reviewed of patients who underwent STA for the treatment of uveitic ME between January 1, 2013, and July 31, 2022, at the University of Colorado Hospital. Uveitic ME was defined by a central subfield thickness (CST) greater than 320 μm or the presence of intra-retinal cystoid spaces on optical coherence tomography (OCT), or by the presence of petaloid macular leakage on fluorescein angiography (FA). Data collected included age, race/ethnicity, sex, history of diabetes mellitus, anatomic classification of uveitis, use of corticosteroids, use of immunomodulatory therapy, presence of intra-retinal fluid on OCT, CST on OCT, and presence of petaloid macular leakage on FA. STA failure was defined as the need for additional therapy within 12 weeks of STA due to persistent or worsening uveitic ME. One hundred eighty eyes from 131 patients were included. Forty-two eyes (23.3%) were considered treatment failures. In univariate and multivariable analysis, higher baseline CST was associated with a higher likelihood of failing STA (OR 1.17 for each 30 μm increase in CST, *P* = 0.016).

**Conclusions:**

STA, while not as potent as intravitreal corticosteroids for the treatment of uveitic ME, was still an effective therapy, particularly for patients with lower baseline CST. Given its lower side effect profile and cost compared to intravitreal treatments, clinicians could consider STA as an initial treatment for mild uveitic ME.

## Background

Macular edema (ME) affects approximately 40% of eyes with uveitis [1, 2]. The presence of ME in uveitis is associated with worse visual prognosis [3]. While uveitis can generally be treated with systemic immunomodulatory therapy (IMT), ME can persist even with adequate control of the intraocular inflammation. For example, in the Multicenter Uveitis Steroid Treatment Trial, 62% of eyes on systemic therapy still required adjunctive local corticosteroid therapy for the treatment of uveitic ME [4].

Uveitic ME can be treated with a variety of medications, including systemic corticosteroids, systemic IMT, topical corticosteroids, and regional corticosteroid injections [5, 6]. Regional corticosteroid injections are commonly used as they avoid the side effects of systemic therapy, while allowing a more constant delivery of medication to the posterior eye without relying on the patient adherence needed for frequent eyedrops. Commonly used injections include sub-Tenon’s triamcinolone acetonide (Kenalog, Bristol-Myers Squibb Company, Princeton, NJ) (STA), intravitreal triamcinolone acetonide (ITA), and the intravitreal 0.7 mg sustained-release dexamethasone implant (Ozurdex, Allergan Inc., Irvine, CA) (IDI). The PeriOcular vs. INTravitreal corticosteroids for uveitic macular edema (POINT) trial compared these three therapies for the treatment of uveitic ME [5]. The trial found that STA was inferior to both intravitreal therapies for treating uveitic ME, using a primary outcome of the proportion of baseline central subfield thickness (CST) at 8 weeks. However, the POINT trial utilized a strict definition of uveitic ME, specifically CST two standard deviations higher than the population normative mean on optical coherence tomography (OCT). This may have lead to the exclusion of eyes with relatively milder ME or ME more prominent on fluorescein angiography (FA) than on OCT. Additionally, STA was still an effective therapy for many patients with uveitic ME in the POINT trial, with approximately 20% of eyes receiving STA having complete resolution of ME at 8 weeks, increasing to 35% at 24 weeks. Other retrospective studies have also demonstrated the utility of STA. For example, Leder et al. reported that 57% of eyes had clinical resolution of uveitic ME 3 months after a single STA [7]. A recent report by Jung et al. evaluating pediatric eyes found that 78% had resolved uveitic ME 3 months after STA [8]. STA also has some advantages over intravitreal corticosteroid therapies, including substantially lower cost, decreased risk of post-injection ocular hypertension [5, 9], no risk for post-injection infectious endophthalmitis, longer duration of action [5, 10–14], and the potential to more safely be delivered via an in-office procedure in the pediatric population [8].

Given these relative benefits, it would be useful for clinicians to know which eyes with uveitic ME may respond well to STA and therefore not require intravitreal therapy. No prior research has evaluated this question. In this study, we aim to identify risk factors predictive for success or failure of STA in the treatment of uveitic ME.

## Main text

### Material and methods

A retrospective chart review was performed on all patients who received STA at the University of Colorado Hospital for treatment of uveitic ME between January 1, 2013, and July 31, 2022, from two uveitis fellowship-trained ophthalmologists (AKR or AGP). The study received approval from the Colorado Multiple Institutional Review Board and all research conformed to the tenets of the Declaration of Helsinki. To reduce the chance that uveitic ME changes were due to systemic medication alterations, patients were excluded if there were changes in systemic IMT in the 8 weeks prior to or 4 weeks after STA, or change in systemic corticosteroids in the 4 weeks prior to STA. Exclusion criteria also included a diagnosis of infectious uveitis, lack of follow-up within 12 weeks following STA, or intraocular surgery within the 12 weeks following STA. Only the first STA that qualified was analyzed for eyes that received multiple STA injections in the study time period.

All injections were done as superior posterior sub-Tenon’s injections, using a modified Nozik technique described previously [8]. This method has been found to have similar efficacy as other periocular routes [15, 16]. Adult patients received 40 mg of triamcinolone acetonide, while pediatric patients received 20 mg.

Uveitic ME was defined by a CST greater than 320 μm [5] or the presence of intra-retinal cystoid spaces using a Heidelberg Spectralis OCT machine (Heidelberg Engineering, Heidelberg, Germany), or by the presence of petaloid macular leakage on wide-field FA in the setting of uveitis. FA was obtained at the discretion of the treating physician.

The following data were collected for each patient eye: age, race/ethnicity, sex, history of diabetes mellitus, history of retinal vein occlusion, laterality of uveitis and injection, dosage of STA, uveitis diagnosis, anatomic classification of uveitis, use of regional corticosteroid injections, use of corticosteroid eyedrops, use of systemic corticosteroids, use of systemic IMT, use of intraocular pressure (IOP)-lowering eyedrops, measures of visual acuity (VA) and IOP, grading of anterior chamber and vitreous cell, presence of intra-retinal and subretinal fluid on OCT, presence of epiretinal membrane on OCT, CST on OCT, and presence of petaloid macular leakage on FA. For eyes with CST greater than 320 μm initially, the CST 12 weeks post-STA injection was also obtained.

STA failure was defined as the need for additional corticosteroid therapy, intravitreal anti-vascular endothelial growth factor injections, or intravitreal methotrexate within 12 weeks of STA due to persistent or worsening uveitic ME, as determined by the treating physician. Corticosteroid therapy included additional corticosteroid eyedrops, local injections, and systemic corticosteroid medications.

#### Statistical analysis

Patients could have one or two eyes included in the study analysis. Demographic and clinical characteristics were summarized for eyes that experience STA success versus failure with basic frequencies and percentages for categorical variables and means, medians, and standard deviations for continuous variables. CST was analyzed both as a continuous variable and categorized into tertiles. The rate of STA failure was calculated for all eyes and by select demographic and clinical variables. Univariate and multivariable logistic regression analysis with generalized estimating equations and an unstructured correlation were used to compare eyes that failed versus eyes that succeeded with STA. Missing data were presented in frequencies and percentages, but were not included in statistical comparisons. Multivariable predictors of STA failure included all variables with *p*-values < 0.10 in univariate analysis with the exception of petaloid macular leakage on FA due to the large number of missing values. Patients with missing CST were also not included in the multivariable model.

### Results

A total of 180 eyes from 131 patients were included in this study (Table 1). Forty-two eyes (23.3%) were considered treatment failures.

**Table 1:** Patient characteristics by STA success or failure by eye

	All Patient Eyes n (column %)	STA Success n (column %)	STA Failure n (column %)	% Failure (Row %)	***P***-value
Number of eyes (row %)	180	138	42	23.3%	–
Mean age, years (SD)	56.0 (17.9)	55.1 (18.8)	59.2 (14.5)	–	0.188
range	9, 90	9, 90	24, 86		
Sex					
Male	74 (41.1%)	61 (44.2%)	13 (31.0%)	17.6%	
Female	106 (58.9%)	77 (55.8%)	29 (69.0%)	27.4%	0.342
Race					
White	101 (56.1%)	82 (59.4%)	19 (45.2%)	18.8%	Reference
Hispanic	26 (14.4%)	23 (16.7%)	3 (7.1%)	11.5%	0.253
Black	44 (24.4%)	27 (19.6%)	17 (40.5%)	38.6%	0.041
Asian^a^	8 (4.4%)	6 (4.4%)	2 (4.8%)	25.0%	0.430
Native American^a^	1 (0.6%)	0	1 (2.4%)	100%	
Anatomic Classification					
Anterior/Anterior and Int.	61 (33.9%)	41 (29.7%)	20 (47.6%)	32.8%	Reference
Intermediate	48 (26.7%)	45 (32.6%)	3 (7.1%)	6.2%	0.005
Posterior	27 (15.0%)	21 (15.2%)	6 (14.3%)	22.2%	0.414
Panuveitis	44 (24.4%)	31 (22.5%)	13 (31.0%)	29.6%	0.808
Presence of diabetes mellitus	35 (19.4%)	23 (16.7%)	12 (28.6%)	34.3%	0.234
Use of systemic corticosteroids	5 (2.8%)	3 (2.2%)	2 (4.9%)	40.0%	0.332
Use of IMT	48 (26.7%)	33 (23.9%)	15 (35.7%)	31.2%	0.136

*STA* sub-Tenon’s triamcinolone acetonide, *SD* standard deviation, *IMT* immunomodulatory therapy

^a^Asian and Native American races combined for statistical comparisons

In the univariate analysis, there were no significant baseline differences between treatment successes and treatment failures with regards to age, sex, presence of diabetes mellitus, use of systemic steroids, use of systemic IMT, or presence of intra-retinal cystoid spaces or subretinal fluid on OCT. In contrast, in the univariate analysis, treatment failures were less likely to have an anatomic classification of intermediate uveitis and more likely to be using topical corticosteroids and have a baseline CST greater than 331 μm (Tables 1 and 2).

**Table 2:** Eye-level characteristics by STA success or failure

	All Patient Eyes n (column %)	STA Success n (column %)	STA Failure n (column %)	% Failure (Row %)	***P***-value
Number of patients (row %)	180	138	42	23.3%	–
Use of topical corticosteroids	99 (55.0%)	69 (50.0%)	30 (71.4%)	30.3%	0.039
Presence of intra-retinal cystoid spaces					
Yes	119 (66.1%)	90 (65.2%)	29 (69.0%)	24.4%	0.604
No	57 (31.7%)	46 (33.3%)	11 (26.2%)	19.3%	
Missing^a^	4 (2.2%)	2 (1.4%)	2 (4.8%)	50.0%	
Presence of sub-retinal fluid					
Yes	21 (11.7%)	14 (10.1%)	7 (16.7%)	33.3%	0.139
No	155 (86.1%)	122 (88.4%)	33 (78.6%)	21.3%	
Missing^a^	4 (2.2%)	2 (1.4%)	2 (4.8%)	50.0%	
CST, um	*n* = 172	*n* = 134	*n* = 38		
Mean (SD)	316 (113)	299 (93.8)	374 (152)		0.002
Median (range)	278 (156, 749)	276 (156, 700)	332 (186, 749)		
CST, tertile category					
156–256	57 (33.1%)	47 (35.1%)	10 (26.3%)	17.5%	0.068
256- < 330	63 (36.6%)	54 (40.3%)	9 (23.7%)	14.3%	0.008
331–749	52 (30.2%)	33 (24.6%)	19 (50.0%)	36.5%	Reference
Petalloid macular leakage on FA					
Yes	127 (70.6%)	103 (74.6%)	24 (57.1%)	18.9%	Reference
No^b^	6 (3.3%)	6 (4.4%)	0	0%	–
No FA	47 (26.1%)	29 (21.0%)	18 (42.9%)	38.3%	0.012

*STA* sub-Tenon’s triamcinolone acetonide, *CST* central subfield thickness, *SD* standard deviation, *FA* fluorescein angiography

^a^Missing data not included in statistical comparisons

^b^No petalloid macular leakage on FA was not included in statistical comparisons due to zero cell size

Within the multivariable analysis, the use of topical corticosteroids (odds ratio [OR], 2.91 [95% CI, 1.07 to 7.95], *P* = 0.037) and increased CST (OR 1.17 for each 30 μm increase in CST [95% CI, 1.03 to 1.32], *P* = 0.016) remained statistically significant for failing STA (Table 3).

**Table 3:** Multivariable predictors of STA failure

	OR (95%CI)	***P***-value
Race		
White	Reference	–
Hispanic	0.32 (0.07, 1.50)	0.150
Black	1.52 (0.58, 3.99)	0.399
Asian/Native American	1.31 (0.19, 9.04)	0.783
Anatomic Classification		
Anterior/Anterior and Int.	Reference	–
Intermediate	0.27 (0.07, 1.08)	0.064
Posterior	0.95 (0.25, 3.58)	0.936
Panuveitis	1.18 (0.32, 4.36)	0.805
Use of topical corticosteroids	2.91 (1.07, 7.95)	0.037
CST, um (for 30 unit change)	1.17 (1.03, 1.32)	0.016

Note: 8 patients with missing CST are not included in the multivariable analysis

*STA* sub-Tenon’s triamcinolone acetonide, *CST* central subfield thickness

**Fig. 1:**
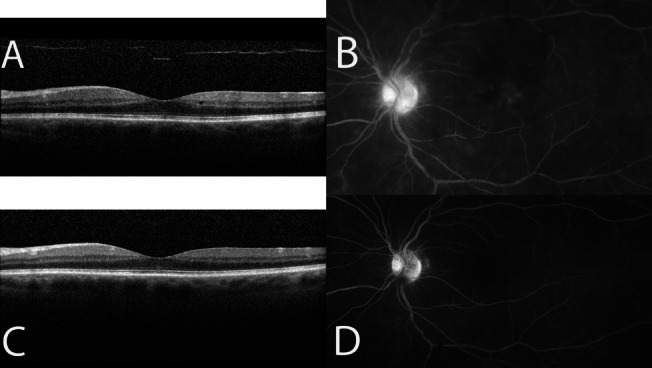
Example of an eye with uveitic macular edema that responded well to sub-Tenon’s triamcinolone acetonide (STA) but would not have met criteria for the POINT trial. The initial OCT macula of the left eye shows intra-retinal cystoid spaces with a central subfield thickness of 258 μm (**a**) and the initial fluorescein angiogram shows petaloid macular leakage (**b**). Two months following STA, the intra-retinal cystoid spaces (**c**) and petaloid macular leakage (**d**) have resolved

Additionally, eyes included in this study that would have met criteria for enrollment in the POINT trial (initial CST greater than 320 μm) were statistically more likely to fail STA (22 of 60 eyes = 36.7%) than eyes that would not have met criteria for POINT trial enrollment (initial CST less than 320 μm; 20 failures out of 120 eyes = 16.7%) (*P* = 0.002). An example of an eye with uveitic ME that responded well to STA but would not have met entry criteria for the POINT trial is illustrated in Fig. 1. The initial OCT macula of the left eye shows intra-retinal cystoid spaces with a CST of 258 μm (Fig. 1a) and the initial FA shows petaloid macular leakage (Fig. 1b). Two months following STA, the intra-retinal cystoid spaces (Fig. 1c) and petaloid macular leakage (Fig. 1d) have resolved.

### Discussion

ME is a common cause of vision loss in uveitis [1]. The treatment of uveitic ME often requires the use of local corticosteroids, either intravitreal (IDI or ITA) or periocular (STA), even in the setting of systemic IMT use [4]. STA has some advantages over intravitreal corticosteroids, including decreased rates of ocular hypertension, increased duration of action, no risk of post-injection infectious endophthalmitis or implant migration, and substantially lower expense [5, 9–14]. However, the POINT trial found that intravitreal corticosteroids were superior to STA in reducing baseline CST 8 weeks following injection in patients with uveitic ME [5]. The POINT trial, though, only included eyes that had a CST greater than 320 μm on the Heidelberg Spectralis or 300 μm on the Zeiss Cirrus (Carl Zeiss AG, Oberkochen, Germany) or Topcon 3DOCT (Topcon, Tokyo, Japan) – two standard deviations higher than the population normative mean – without consideration for the presence of intra-retinal cystoid spaces on OCT or petaloid macular leakage on FA. While this strict cut-off increases the specificity for ME, it likely selects for eyes with more severe ME and excludes eyes that have ME by parameters other than CST.

Our study indicates that eyes with more severe ME, as defined by a higher CST, are more likely to fail STA for the treatment of uveitic ME. Specifically, each 30 μm increase in CST corresponded to a 17% increased likelihood of failing STA. Similarly, eyes that would have met criteria for the POINT trial were more likely to fail STA than eyes that would not have met criteria for the POINT trial (CST less than 320 μm). Correlating with this, when CST data is segmented into tertiles, eyes in the highest tertile (CST of 331–749 μm) were more likely to fail STA than eyes in the bottom two tertiles. However, it should be noted that over 60% of eyes in this highest tertile for CST still responded favorably to STA in our study.

The use of topical corticosteroids was also associated with an increased likelihood of failing STA in univariate and multivariable analysis. This could be an additional indicator of more severe ME, as the use of topical corticosteroids was at the discretion of the treating physician, and eyes with more severe inflammation and/or ME may have been more likely to be prescribed this additional anti-inflammatory therapy.

The limitations of this study include its retrospective nature, variability of baseline uveitic characteristics and prior treatments, absent data for some variables, and potential patient selection bias, as patients with milder uveitis overall may have been more likely in our practice to have received STA.

### Conclusions

Overall, our data suggests that eyes with less retinal thickening secondary to uveitic ME may do well with STA and not require intravitreal therapy. While intravitreal corticosteroids are clearly very effective for uveitic ME, they do have some relative disadvantages compared to STA, of which one of the most important is the substantially higher cost. The preservative-free triamcinolone acetonide required for intravitreal use is approximately four times more and the IDI approximately 70 times more expensive than the triamcinolone acetonide used for periocular injections [17]. In the setting of rising costs of healthcare generally, and intravitreal injections specifically [18], it would be reasonable for clinicians to consider STA as an initial treatment for mild uveitic ME, and then advance to intravitreal therapies as needed.

## Data Availability

The datasets used and/or analyzed during the current study are available from the corresponding author on reasonable request.

## Abbreviations

ME: Macular edema
IMT: Immunomodulatory therapy
STA: Sub-Tenon’s triamcinolone acetonide
ITA: Intravitreal triamcinolone acetonide
IDI: Intravitreal 0.7 mg sustained-release dexamethasone implant
POINT: PeriOcular vs. INTravitreal corticosteroids for uveitic macular edema trial
CST: Central subfield thickness
OCT: Optical coherence tomography
FA: Fluorescein angiography
IOP: Intraocular pressure
VA: Visual acuity
OR: Odds ratio
